# Chlorogenic Acid: A Systematic Review on the Biological Functions, Mechanistic Actions, and Therapeutic Potentials

**DOI:** 10.3390/nu16070924

**Published:** 2024-03-23

**Authors:** Vi Nguyen, Elaine G. Taine, Dehao Meng, Taixing Cui, Wenbin Tan

**Affiliations:** 1 Department of Cell Biology and Anatomy, School of Medicine, University of South Carolina, Columbia, SC 29209, USA; vi.nguyen@uscmed.sc.edu; 2 TritaliMed, Inc., Columbia, SC 29223, USA; 3 Applied Physics Program, California State University San Marcos, San Marcos, CA 92096, USA; 4 Dalton Cardiovascular Research Center, Department of Medical Pharmacology and Physiology, School of Medicine, University of Missouri, Columbia, MO 65211, USA; taixingcui@health.missouri.edu; 5 Department of Biomedical Engineering, College of Engineering and Computing, University of South Carolina, Columbia, SC 29208, USA

**Keywords:** chlorogenic acid, inflammation, oxidation, metabolic homeostasis, AMPK, NF-kB, neuromodulation, chronic metabolic diseases

## Abstract

Chlorogenic acid (CGA) is a type of polyphenol compound found in rich concentrations in many plants such as green coffee beans. As an active natural substance, CGA exerts diverse therapeutic effects in response to a variety of pathological challenges, particularly conditions associated with chronic metabolic diseases and age-related disorders. It shows multidimensional functions, including neuroprotection for neurodegenerative disorders and diabetic peripheral neuropathy, anti-inflammation, anti-oxidation, anti-pathogens, mitigation of cardiovascular disorders, skin diseases, diabetes mellitus, liver and kidney injuries, and anti-tumor activities. Mechanistically, its integrative functions act through the modulation of anti-inflammation/oxidation and metabolic homeostasis. It can thwart inflammatory constituents at multiple levels such as curtailing NF-kB pathways to neutralize primitive inflammatory factors, hindering inflammatory propagation, and alleviating inflammation-related tissue injury. It concurrently raises pivotal antioxidants by activating the Nrf2 pathway, thus scavenging excessive cellular free radicals. It elevates AMPK pathways for the maintenance and restoration of metabolic homeostasis of glucose and lipids. Additionally, CGA shows functions of neuromodulation by targeting neuroreceptors and ion channels. In this review, we systematically recapitulate CGA’s pharmacological activities, medicinal properties, and mechanistic actions as a potential therapeutic agent. Further studies for defining its specific targeting molecules, improving its bioavailability, and validating its clinical efficacy are required to corroborate the therapeutic effects of CGA.

## 1. Introduction

Chlorogenic acid (CGA) family members are abundant dietary phenolic acid compounds in plants, conjugating the hydroxy group of quinic acid and the carboxyl group of caffeic acid as the parent structure. CGA family includes (1) 1L-(−)-quinic acid, (2) caffeic acid (CA), (3) ferulic acid, and (4) the p-coumaric acid (p-CoQA) group including p-CoQAs, caffeoylquinic acids (CQAs), and feruloylquinic acids (FQAs) [1,2,3,4]. The CGA family has shown multiple protective effects on mitigating many chronic inflammatory and age-related disorders through exerting the central actions of anti-inflammation, antioxidation, and metabolic homeostasis modulation [1,2,3,4].

CGA has limited bioavailability in plant foods due to the esterification with cell wall components such as proteins, lignin, and cellulose [5]; thus, appropriate food processing is needed to facilitate release [6]. CGAs are enriched in green coffee bean extract (GCE), which may comprise 54% of its contents [7]. Particularly, 5-CQA and 3-CQA present about 35−40% and 10−15% among CGA components, respectively [8]. The roasting process leads to a dramatic decrease in the total amount of CGAs and changes in CGA compositions with main contents of 3,4-di-CQA, 5-CQA, 4-CQA, and 3-CQA [9]. One-third of CGA is metabolized quickly after direct absorption in the upper gastrointestinal tract upon oral administration [3,10]. The esterase secreted by the intestine microbes (such as *Lactobacillus gasseri, Bifidobacterium lactis*, and *Escherichia coli*) can hydrolyze the remaining CGAs and release CGA and quinic acid to be absorbed in the intestines [11,12].

CGA exhibits a good safety profile, which has not shown any obvious adverse effect and toxicity to normal cells or tissues, and is well-tolerated by humans [13,14]. In an acute toxicity experiment, no side effects are observed in mice for two weeks upon an intake of CGA-enriched GCE (1 g/kg) [15]. A single dose of GCE (2 g/kg) (containing 50% CGA) in rats does not cause any type of toxicity. Rats with an intake of CGA (250, 500, and 1000 mg/kg) show no adverse effects in three months [16]. Cautiously, a high-dosage consumption of CGA (2 g/day) or black tea (4 g/4 L/day) four times in 7 days can moderately increase plasma homocysteine levels by 12% or 11% in humans, respectively [17].

In this review, the literature search was conducted between 2005 and 2024 in the database of PubMed for articles related to the subjects using the specific keywords of “chlorogenic acid” and (“inflammation” or “oxidation”). Inclusion criteria included full-text publications in English. Exclusion criteria included preprints and extracts without mention of CGA as the active ingredient in the abstract. The publications in this search result served as core literature for this review.

## 2. Functional Hubs of CGA’s Pharmacological Effects

### 2.1. Anti-Inflammation and Anti-Oxidation (Figure 1A)

Multidimensional effects of CGA in multiorgan are exerted through or related to its anti-inflammation and anti-oxidation properties. CGA has shown compelling immunomodulatory effects for mitigating pathological developments related to inflammatory response and/or oxidative stress [18]. The mechanisms underlying the anti-inflammation properties of CGA are multi-dimensional. First, it attenuates pathogen-activated nuclear factor-κB (NF-κB), c-Jun N-terminal kinase (JNK), extracellular signal-regulated kinases (ERK), and p38-mitogen-activated protein kinase (MAPK) signaling pathways [19,20,21,22,23] (Figure 2A). Second, it inhibits the synthesis of many pro-inflammatory factors such as tumor necrosis factor-α (TNF-α), interleukin 1 beta (IL-1β), IL-6, interferon-γ, monocyte chemotactic protein-1, and macrophage inflammatory protein-1α during the inflammatory response [24,25,26] (Figure 2A). CGA could suppress TNF-α-induced inflammatory and oxidative stress in the pre-adipocyte 3T3-L1 cell line [27]. In lipopolysaccharide (LPS)-treated RAW264.7 cells, CGA inhibits cyclooxygenase-2 (COX-2) upregulation and suppresses the release of PGE2.19. Third, it reduces Toll-like receptor (TLR) activity and modulates the release of cytokine and chemokine, thus suppressing sepsis-induced pathologies [28,29,30,31]. CGA could counteract LPS-induced inflammation and oxidation by activation of the CD36/AMPK/PGC-1alpha pathway in RAW264.7 macrophages [32]. Intraperitoneal administration of CGA decreases neutrophilic infiltration by counteracting LPS-induced TLR-4, TNF-α, and NF-κB signaling in mouse liver [28]. CGA inhibits the systemic accumulation of high-mobility group box 1 (HMGB-1) and prevents sepsis-induced mortality [29,33]. The antioxidative activities of CGA are related to the activation of nuclear factor erythroid 2-related factor 2 (Nrf2)-dependent or -independent pathways as well as its anti-inflammatory properties [20,34,35,36,37] (Figure 2B). CGA effectively eliminates free radicals and inhibits oxidative injuries and apoptosis in multi-tissues by suppressing caspases’ activities [20,34].

**Figure 1:**
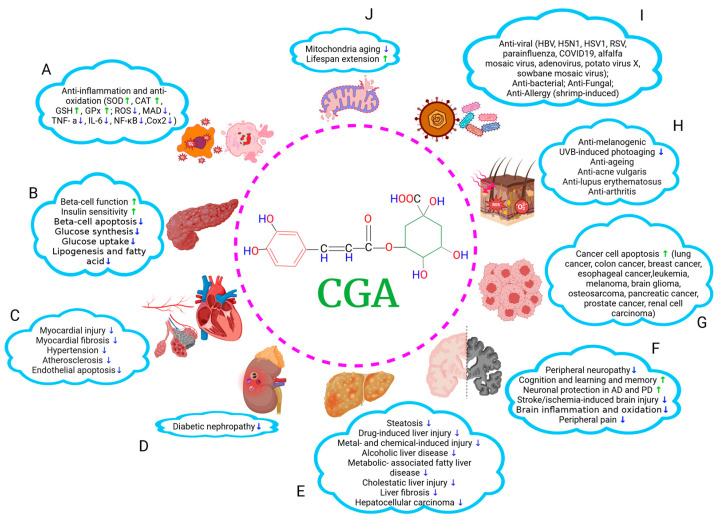
A summary of therapeutic effects of CGA on multiorgan. CGA shows various beneficial roles in many pathological conditions. It can (**A**) mitigate inflammatory response and oxidative stress; (**B**) modulate glucose and lipid homeostasis and alleviate DMs; (**C**–**E**) protect cardiovascular system, kidneys, and liver; (**F**) facilitate the recovery from neurological impairments such as neurodegenerative disorders and diabetic peripheral neuropathy; (**G**) inhibit tumor cell proliferation and migration; (**H**) ameliorate skin pathologies; (**I**) execute anti-pathogen effects, and (**J**) exert antiaging effects. ↑, increasing; ↓, decreasing. The graph was created with Biorender.com.

**Figure 2:**
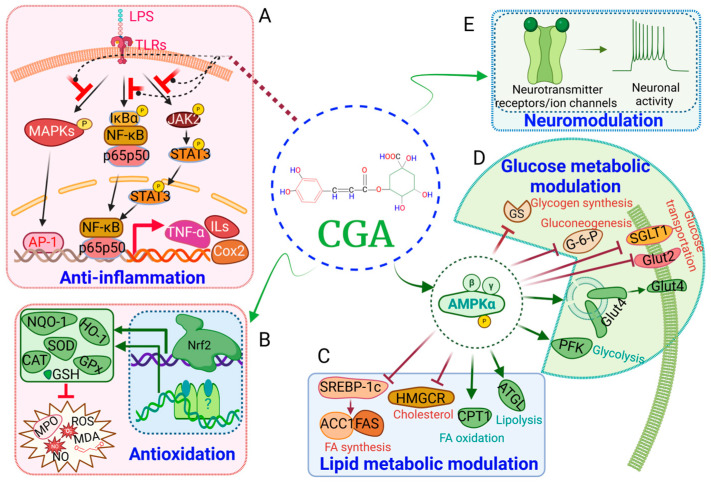
The potential mechanistic actions of CGA on multi-targets. CGA can potentially (**A**) target NF-kB, MPAKs, and JAK pathways to mitigate inflammation; (**B**) activate Nrf2-dependent and independent pathways to execute antioxidation function; (**C**) regulate lipid metabolism through increasing lipolysis and fatty acid oxidation and suppressing synthesis of cholesterol and fatty acids; (**D**) modulate glucose metabolism through increasing glycolysis and suppressing glucose uptake and glucose synthesis; and (**E**) exhibit neuromodulation through targeting multiple neuroreceptors and ion channels. The graph was created with Biorender.com.

### 2.2. Glucose and Lipid Metabolic Homeostasis Modulation (Figure 1B)

CGAs can facilitate the maintenance of metabolic homeostasis of glucose and lipids [38]. One mechanism involves the modulation of activities of AMP-activated protein kinase (AMPK) and ERK1/2 [4] (Figure 2C,D). AMPK is a master energy sensor that regulates cellular glucose and lipid metabolism. These functions directly underlie the CGA effects on the mitigation of chronic metabolic-associated syndromes such as obesity, diabetes mellitus (DM), and their complications. CGA or CGA-containing extracts can inhibit pancreatic lipase activity [39]. CGA exhibits inhibitory effects on the function of many lipid metabolic enzymes including fatty acid synthase, HMG-CoA reductase, and cholesterol acyltransferase in mice fed on a high-fat diet (HFD) [40] (Figure 2C). CGA can upregulate AMPK and carnitine palmitoyltransferase I (CPT-1) and inhibit acetyl-CoA carboxylase (ACC), thus reducing hepatic and blood levels of triglyceride (TG) and free fatty acids (FFA) in HFD rats [41] (Figure 2C). CGA can facilitate cholesterol elimination by modulating homeostasis of bilirubin and bile acids via farnesoid X receptor (FXR) and peroxisome proliferator-activated receptor (PPAR) gamma coactivator 1-alpha (PGC-1α) or fibroblast growth factor (FGF) 15 pathways [42,43].

CGA can attenuate glucose absorption. CGA can decrease sodium–glucose co-transporter 1 (SGLT-1), thus reducing glucose uptake and causing reduced glucose-dependent insulinotropic polypeptide (GIP) release and altered gut microbiota profile [44]. CGA activates AMPK pathways and suppresses HFD-induced upregulation of SGLT-1, glucose transporter type 2 (GLUT-2), and proglucagon (Plg), leading to an increase in the translocation of GLUT4 to plasma membranes and inhibition of liver glucose production [45,46] (Figure 2D). Activation of AMPK may also be prompted by caffeic acid, a metabolite of CGA, for modulating glucose transport [47].

CGA can reduce glucose release. CGA can inhibit glucose-6-phosphatase (G6Pase), the enzyme converting glycogen to glucose [44]. CGA inhibits the expression and activity of hepatic *α*-glucosidase and G6Pase, reduces the hydrolysis of hepatic glycogen, and activates AMPK pathways, resulting in attenuation of hepatic steatosis and improvements of metabolic indexes including fasting serum glucose (FSG) level, glucose tolerance, glucose uptake, insulin sensitivity, and lipid profiles [38,48,49] (Figure 2D).

CGA can modulate plasma levels of glucose and lipids. In HFD golden hamsters or rats, CGA upregulates hepatic PPAR-α levels, increases the activity of hepatic lipase (HL), decreases hepatic levels of TG and FFA and fasting serum levels of TG, FFA, total cholesterol (TC), low-density lipoprotein cholesterol (LDL-c), high-density lipoprotein cholesterol (HDL-c), FSG, and insulin (FSI), as well as attenuates the activity of lipoprotein lipase (LPL) in skeletal muscle [50,51]. CGA-containing GCEs (100 mg/kg, 6 weeks) can decrease blood glucose levels, body weight, and fat mass in mice fed on an HFD [52]. CGA (oral gavage 80 mg/kg/day, 12 weeks) in db/db mice can lower FSG, adiponectin, and TG, and increase muscle glycogen via up-regulating hepatic PPAR-α and inhibiting G6Pase expression [53]. Post-meal CGA treatment (60 min) decreases the level of blood sugar compared to the placebo in rats [54].

### 2.3. Human Subject Studies

In a randomized crossover study, healthy postmenopausal women (BMI 25–40, *n* = 16) with consumption of the bioactive yogurt containing curcumin and CGA showed significantly lower plasma levels of TNFα compared to the placebo group and the baseline [55]. In an acute pilot study, healthy subjects (*n* = 31) were given a single dose of a polyphenol-rich beverage (PRB) or placebo. The plasma levels of 8-iso-PGF2-alpha and advanced oxidation protein products were decreased, and hydroxyl radical antioxidant capacity at one-hour post intake of PRB was increased compared to the baseline [56].

In a cohort of 15 patients with impaired glucose tolerance (IGT), CGA (400 mg three times per day for 3 months) decreased FSG, insulinogenic index, body weight, body mass, waist circumference, TG, TC, LDL-c, and very low-density lipoprotein levels, with an upregulated Matsuda index [57]. In a randomized, double-blind controlled trial, participants (*n* = 65) were given an 8-week cooked ham enriched with a pool of antioxidants (including 22.5 mg CGA/100 g cooked ham) or received a placebo. Subjects with intervention showed significantly lower levels of ox-LDL, malondialdehyde (MDA), TC, high-sensitive C-reactive protein (hs-CRP), and IL-6 [58]. In a cohort of overweight dyslipidemic subjects (*n* = 90), a nutraceutical (containing bergamot, phytosterols, vitamin C, and CGA) or placebo was administered for 8 weeks. The subjects with the treatment showed improved lipid and glucose metabolism, which were associated with reduced levels of TG, LDL-c, non-HDL-c, the ratio of leptin/adiponectin, hs-CRP, and TNFα [59]. In a randomized, cross-over, controlled study, hypercholesterolemic subjects (*n* = 27) were administered soluble green/roasted (35:65) coffee or placebo for 8 weeks. The subjects showed lower lipid parameters (TC, TG, LDL-c, VLDL-c), MDA, and protein carbonyl group oxidation, systolic and diastolic blood pressures (SBP, DBP), heart rate, and body weight compared with the baselines [60]. Habitual coffee intake decreased serum levels of IL-18 and 8-isoprostane but increased adiponectin and HDL-c in healthy subjects (*n* = 47) [61]. In a study, healthy, overweight subjects (*n* = 142, BMI ≥25 to <30 kg/m^2^) were given a high-CGA (369 mg CGA/serving) or control (35 mg CGA/serving) coffee for 12 weeks. subjects with an intake of high-CGA coffee showed significant improvements in lowering the visceral fat area (VFA), total abdominal fat area (TFA), BMI, and waist circumference compared to those in the control group [62]. In a cohort of 21 patients with metabolic syndrome, CGA-containing GCE (400 mg twice per day for 2 months) showed a decrease in levels of FSG, insulin resistance, weight, and BMI in patients [8]. In a study of healthy Japanese women (*n* = 57), plasma CGA showed a negative association with FSG, glycated hemoglobin, and CRP [63].

CGAs showed diverse effects on neuroprotection for neurodegenerative disorders and diabetic peripheral neuropathy, mitigation of cardiovascular disorders, skin diseases, diabetic mellitus, liver and kidney injuries, and anti-tumor activities (Figure 1). Mechanistically, their anti-inflammation and anti-oxidation properties and metabolic modulations underlie these pharmacological activities for protection against cell injuries, restoration of cellular function, and maintenance of physiological and metabolic homeostasis (Figure 2), which is discussed across various tissues and disorders in this review.

## 3. Cardiovascular Protective Effect

CGA can exert protective roles at multiple levels in various cardiovascular complications, including mitigating hypotension, improving endothelial cell function, alleviating atherosclerosis, and ameliorating cardiomyopathy.

### 3.1. Hypotensive Effects (Figure 1C)

CGA can function as a hypotensive agent to lower blood pressure in a dose-dependent manner in spontaneously hypertensive rats (SHRs) [64,65]. Mechanistically, CGA-mediated vasodilation can occur through suppressing the activity of NADPH oxidase (NOX), inhibiting the generation of radical oxygen species (ROS), and increasing nitric oxide (NO), thus mitigating endothelial dysfunction in SHRs [64]. Particularly, the nitric oxide synthase (NOS), COX, and endothelium-derived hyperpolarizing factor (EDHF) pathways are involved in CGA-mediated vasodilation [66]. Furthermore, HPT-related pathogenic factors, including angiotensin-converting enzyme (ACE), arginase, and cholinesterase, are suppressed in cyclosporine-induced HPT rats upon administration of CGA for one week, suggesting the hypotensive and cardioprotective effects of CGAs [67]. Collectively, CGAs can ameliorate HPT by enhancing vasodilation, mitigating endothelial dysfunction, and reducing vascular remodeling triggered by hypoxia. The underlying mechanisms are related to an elevation of EDRFs and associated enzymes (NO, PGI_2_, Ach, NOS, and COX), suppression of oxidations (ROS and NOX) and vasoconstrictors (arginase, ANG II, cholinesterase, and ACE), decrease in levels of hypoxia-inducible factor 1α (HIF-1α) and phosphorylated c-Src, and an enhancement of Shc/Grb2/ERK1/2 signaling [2,64,66,67].

### 3.2. Effects of Endothelial Protections and Anti-Atherosclerosis (Figure 1C)

CGA can inhibit the oxidation of LDL and the subsequent endothelial damage caused by oxidized LDL (ox-LDL). CGAs can lower blood lipid levels [68,69,70]. CGA inhibits Cu^2+^-induced LDL oxidation [71,72]. Paraoxonase 1 (PON1) is an esterase that inhibits the formation of oxidized lipoproteins (ox-LDL and ox-HDL) [73]. CGA can protect PON1 from inactivation, thus suppressing the generation of ox-LDL [74]. CGA upregulates sirtuin 1 (SIRT1) and AMPK/PGC-1 activity, thus protecting mitochondrial function and suppressing ox-LDL-caused endothelial injuries [75]. CGA suppresses the levels of transient receptor potential canonical channel 1 (TRPC1) and decreases ROS and Ca^2+^, thus mitigating lysophosphatidylcholine (LPC)-induced endothelial injuries [69,76]. CGA protects endothelial cells by reducing ROS, xanthine oxidase-1, and HOCl-induced oxidative damage, enhancing superoxide dismutase (SOD), and producing NO and heme oxygenase (HO)-1 [77,78]. CGA modulates mtROS/JNK/NF-κB signaling, thus inhibiting inflammation and regulating mitochondrial bioenergetics in hearts. Moreover, CGA can regulate ankyrin-B levels as a cardiomyocytic defender [2].

CGAs can alleviate atherosclerosis by inhibiting endothelial damage, platelet–leukocyte interactions, and the levels of adhesion molecules, as well as upregulating prometabolic and antiplatelet pathways [2]. CGA inhibits adhesion molecules in early atherosclerosis, including IL-1β and TNFα-induced vascular cell adhesion molecule-1, intercellular cell adhesion molecule-1, and endothelial selectin; it also blocks α-glucosidase activities in human endothelial cells [79,80,81]. CGA suppresses hypoxia-induced HIF-1α-VEGF signaling, thus blocking angiogenesis and mitigating atherosclerosis [82]. Furthermore, CGA can inhibit VEGF-induced endothelial proliferation and migration by modulating VEGFR2, ERK ½, and protein kinase B (Akt) signaling [83]. CGA prevents platelet aggregation in the atherothrombotic process via the A_2A_ receptor/adenylate cyclase (AC)/cAMP/protein kinase A (PKA) pathway [84,85]. CGA (400 mg/kg/day) can decrease the lesional areas of atherosclerosis in ApoE^−/−^ mice by activating the PPARγ–liver X receptor α (LXRα)–ATP-binding cassette transporter A1 (ABCA1) signaling [86,87].

### 3.3. Cardioprotective Effects (Figure 1C)

Myocardial injuries can be triggered by TNF-α signaling which is activated by MAPKs such as p38 and JNK/SAPK and NF-κB pathways [88,89]. CGAs can regulate NF-κB and PPARα pathways, lower HIF-1α expression, and suppress cardiac apoptotic signaling, thus executing beneficial effects against cardiac hypertrophy and heart failure (HF) [2]. In a transverse aortic constriction (TAC)-induced HF mouse model and a TNF-α-induced pluripotent stem cell-derived cardiomyocyte injury model, CGA can inhibit NF-κB and JNK pathways, exhibiting cardioprotection [90]. CGA inhibits cardiomyocytic hypertrophy by upregulating IκBα and suppressing NF-κB to be translocated into the nucleus [91]. CGA-enriched chrysanthemum extract (CME) prevents myocardial hypertrophy by targeting HIF-1α and PPARα pathways in rats with renal hypertension and H9C2 cells with stimulation of ANG II-hypoxia [92]. CGAs have been shown to effectively protect from peroxidation of heart membranes and cardiac mitochondria [93,94,95]. Moreover, 3,5-di-CQA shows inhibition of myocardial injuries through increasing the activity of phosphatidylinositol 3-kinase (PI3K)/Akt in *tert*-butyl hydroperoxide (TBHP)-treated H9C2 cells [96].

CGAs can mitigate the inflammations, oxidations, defects of mitochondrial respiration, lysosomal dysfunction, and apoptosis, showing alleviative roles in multiple myocardial infarction (MI) models, including the animal models induced by ISO, left anterior descending coronary artery (LAD), and carbon tetrachloride (CCl_4_), as well as LAD-induced myocardial ischemia/reperfusion (I/R) senescence-accelerated prone 8 (SAMP8) mouse [97,98,99,100,101]. CGA-enriched extracts from *Erigeron multiradiatus* (Lindl.) Benth. inhibit NF-κB and JNK activation and suppress myocardial leukocyte infiltration and inflammatory response, thus alleviating acute MI in rats after a single administration intravenously (10, 20, and 40 mg/kg) [102].

### 3.4. Human Subject Studies for Cardiovascular Protection

CGA could lower SBP and DBP in patients with mild hypertension [7,14,103,104]. For example, Kozuma et al. showed that daily oral ingestion of GCE (93 or 185 mg for 4 weeks) could lead to a reduction of 4.7 and 5.6 mmHg in levels of systolic blood pressure (SBP) and a decrease of 3.3 and 3.9 mmHg in levels of diastolic blood pressure (DBP), respectively, in patients with hypertension [7]. In a randomized trial in Japanese patients with mild essential hypertension (HPT), CGA (140 mg/day) for 12 weeks could lower 10 mmHg of SBP and 6 mmHg of DBP [14]. Mild HPT patients taking CGA (228 mg/day for 1 month) showed a reduction of 3.3 and 2.8 mmHg in levels of SBP and DBP, respectively [104]. Ferulic acid is considered one of the active substances of CGA for producing a strong hypotensive effect via muscarinic acetylcholine receptors after short- and long-term ingestions [64,65]. In a clinical trial of patients with borderline or stage 1 hypertension (*n* = 37), a single intake of coffee with a high content of CGAs and low content of hydroxyhydroquinone (HHQ) significantly improved postprandial flow-mediated vasodilation and decreased circulating 8-isoprostane levels, which was effective for improving postprandial endothelial dysfunction [105]. A separate study showed that healthy male adults with ingestion of CGA without HHQ for four weeks could significantly increase postprandial fat oxidation and the ratio of postprandial biological antioxidant potential (BAP) to the derivatives of reactive oxygen metabolites (d-ROMs) compared to those with an intake of CGA with HHQ [106]. CGAs can incorporate specific phenolic acids into LDL particles to lower the risk of their oxidations in human subjects [70,71,107].

In a randomized controlled trial, healthy adults with an 8-week consumption of CGA-enriched coffee beverages showed a significant decrease in levels of twelve urine oxylipins compared to the baseline. Oxylipins are generated during foam cell formation in atherogenesis and thus are biomarkers for CVDs [108]. In a cohort of healthy subjects (*n* = 25), the impact of consumption of coffee containing 787 mg or 407 mg CGAs on CVD risk markers such as oxysterols and FFAs was assessed. Subjects with an intake of coffee showed a decrease in oxysterols and FFAs and an increase in cholesteryl esters. While subjects in the placebo group showed an elevation of oxysterols and FFAs and a reduction in cholesteryl esters [109]. Healthy subjects with consumption of decaffeinated GCE (CGAs accounting for about 51.2% constituents) showed an acute improvement in flow-mediated dilation (%FMD) of the brachial artery [110]. A study from two randomized trials with healthy male subjects (*n* = 15) showed that coffee intake could acutely improve human vascular function, likely through 5-CQA and its physiological metabolites [111]. A higher response of FMD induced by CGA-rich coffee was also reported in a study with 12 healthy subjects [112]. Healthy adults with an intake of a coffee berry beverage (containing 440 mg chlorogenic acid) could increase subjective energic arousal and hemodynamic responses from cerebral blood flow compared with the baseline [113]. In a placebo-controlled double-blind pilot study with healthy Japanese men (*n* = 16), subjects with the intake of GCE showed significantly greater changes in cardio-ankle vascular index (CAVI) (e.g., increasing FMD and decreasing sympathetic nervous activity) than those in the placebo group [114]. In a randomized, double-blind, placebo-controlled study, subjects (*n* = 50, BMI ≥ 25 to <30 kg/m^2^) were given a nutraceutical containing CGA and luteolin extracts for 6 months. Participants in the treatment group showed significantly decreased body weight, glycemic and lipid parameters (TC, TG, LDL-c) as well as improved hepatic functionality, carotid-media thickness (CIMT), and endothelial function compared to the subjects in the placebo group [115]. In a separate study of subjects with metabolic syndrome (*n* = 50), a 6-month intake of the same nutraceutical significantly improved hepatic and cardio-metabolic parameters in the patients [116].

## 4. Mitigative Effects on Diabetes Mellitus (DM)

CGA has shown its functions in protecting β cells from apoptosis, improving β cell function, facilitating glycemic control, and mitigating DM complications.

### 4.1. Protective Effects on β Cells (Figure 1B)

CGA can competitively reduce *α*-amylase activity [81,117]. CGA shows inhibition on porcine pancreatic *α*-amylase (PPA), PPA-I, and PPA-II [118]. CGA can enhance insulin secretion in *β* cells and Langerhans from rat islets [119,120]. CGA can reduce obesity-related insulin resistance in mice fed on HFD or high-fat milk, spontaneously obese mice, or rats fed on HFD [121,122,123]. One mechanism underlying CGA’s effects on decreasing insulin resistance and increasing insulin sensitivity is related to antioxidative stress. CGA reduces levels of lipid hydrogen peroxide and increases plasma antioxidants such as glutathione (GSH), vitamin C, vitamin E, and ceruloplasmin in DM model rats [124]. CGA scavenges thiobarbituric acid reactive substances and hydroperoxide through upregulation of SOD, catalase (CAT), glutathione peroxidase (GSH-Px), and glutathione S-transferase (GST) in the liver and kidney [125]. CGA suppresses inflammatory response by downregulation of F4/80+ and CD68+ macrophages in the liver and white adipose tissues [121]. CGA increases GSH and GSH-Px and reduces ROS, thus protecting *β* cells from exposure to streptozotocin (STZ) [120]. In STZ-induced DM rats, CGA (5 mg/kg/day, 45 days) in combination with tetrahydrocurcumin (80 mg/kg/day, 45 days) can mitigate the STZ-induced aberrances of enzymes related to gluconeogenesis (G6Pase and fructose-1,6-bisphosphatase) and glycolysis (glucokinase and hexokinase), thus lowering the levels of blood glucose and glycosylated hemoglobin (HbA(1C)) and elevating the levels of insulin, C-peptide, hemoglobin, and glycogen [126].

### 4.2. Mitigative Effects on DM Complications (Figure 1D)

CGA reduces glomerular hypertrophy and proliferation and mesangial cell expansions, decreases kidney malondialdehyde (MDA) levels, increases antioxidants (such as SOD, CAT, and GSH-Px), and reduces factors associated with oxidation and inflammation (such as IL-6, TNF-α, COX-2, and IL-1β) in the kidney of a diabetic nephropathy rat model [127,128]. CGA-containing extracts suppress vascular proliferation in kidneys induced by STZ and decrease serum VEGF levels induced by HIF-1α in DM mice [129,130]. In a diabetic retinopathy rat model, CGA shows restoration of the impaired tight junction protein occludin, mitigation of aberrant retinal vascular permeability, and protection of the integrity of the blood–retinal barrier [131]. In DM mice, CGA alleviates diabetic peripheral neuropathy (DPN)-induced auditory dysfunction by functional restoration of cochlear hair cells and protection of the external auditory canal [132]. CGA can relieve DM-induced neuropathic pain [133].

### 4.3. Human Subject Studies for Glycemic Control

CGA can attenuate FSG and insulin production in patients [57]. In a randomized, double-blind, placebo-controlled crossover study to evaluate acute response, a one-time intake of green tea catechins (GTC) together with coffee CGA significantly increased GLP-1 and decreased blood sugar levels and GIP secretion in healthy subjects compared with the placebo group after consumption of a 75 g glucose load [134]. This data was echoed by a related study showing that a three-week intake of GTC + CGA-enriched beverages exhibited similar beneficial effects in postprandial glycemic control and diabetic prevention [135]. In a cohort of subjects with prediabetic impaired fasting glucose (IFG), CGA-rich *Cynarascolymus* (Cs) extracts (*n* = 27) or placebo (*n* = 27) were administered. The subjects in the treatment group showed significant improvements in glycemic control, insulin sensitivity, and many metabolic parameters (TC, LDL-c, HDL-c, TG, ApoA, ApoB, and glycated hemoglobin) [136]. In a randomized clinical trial in patients with metabolic syndrome, participants with an intake of GCE (400 mg, twice per day, 8 weeks) significantly decreased SBP, FBS, homoeostatic model of assessment of insulin resistance, waist circumference, and appetite scores in comparison to those in the placebo group [8].

## 5. Hepatoprotection

CGA can mediate hepatoprotective roles in various pathological conditions of the liver via antioxidant and anti-inflammatory features [4]. (1) It can inhibit TLR4-mediated activation of NF-κB, thus suppressing pro-inflammatory responses; (2) it can activate the AMPK pathway to modulate metabolic homeostasis; (3) it can increase the activity of the Nrf2 pathway, thus exerting antioxidant effects; and (4) it can inhibit caspases’ activation to suppress hepatic apoptosis induced by chemicals or toxins.

### 5.1. Hepatoprotection from Metal-, Chemical-, Drug-, and Toxin-Induced Liver Injury (Figure 1E)

CGA can activate Nrf2 and inhibit the TLR4/NF-κB signaling cascade, reduce activities of serum liver enzymes, oxidation, and inflammation, and alleviate liver injuries caused by the following metals and chemicals: sodium arsenite [137], lead (Pb) [138], cadmium (Cd) [139], aluminum chloride [140], polychlorinated biphenyls [141], TAA [142], carbon tetrachloride (CCl4) [143], D-gal [144], L-carnitine [145], lipopolysaccharide (LPS) [146,147], palmitic acid [148], and aflatoxin B1 [149].

CGA mitigates acetaminophen-induced hepatic injuries by inhibiting apoptosis and oxidation, ameliorating liver inflammation, activating Nrf2, promoting mitophagy, and suppressing activities of metabolic enzymes such as cytochrome P450 (CYP) [20,24,150,151,152,153,154]. CGA can ameliorate hepatotoxicity triggered by many other drugs including tamoxifen, methotrexate, triptolide, and monocrotaline [155,156,157,158].

CGA can attenuate alcohol-induced pathologies such as steatosis, apoptosis, and fibrosis by regulating CYP2E1/Nrf2 and TLR4/NF-κB [159], scavenging mitochondrial and intracellular ROS [160], and facilitating n-butyric acid generation for homeostatic regulation of the gut–liver axis [161].

### 5.2. Mitigative Effects on Metabolic-Associated Fatty Liver Disease (MAFLD) (Figure 1E)

CGA inhibits HMG-CoA reductase, thus reducing the quantity of palmitic acid, oleic acid, or linoleic acid-induced large lipid droplets in the hepatic cell line HepG2 [162,163]. CGA attenuates MAFLD in HFD mice by increasing the production of glucagon-like peptide-1, reducing ER stress, suppressing mucosa barrier injury in the intestine, and inhibiting JNK signaling, as a result of autophagic suppression and insulin-resistant mitigation [123,164,165].

CGA in combination with metformin [166] or geniposide [167,168,169,170] improves MAFLD through multiple mechanisms. CGA combined with telmisartan improves rat MAFLD caused by high fructose, possibly through suppressing sphingosine kinase 1 (SPHK-1)/sphingosine-1-phosphate/TLR4 pathways [171]. Lipid metabolism is modulated by CGA in combination with caffeine via the AMPKα-LXRα pathway in HFD-fed mice [172]. CGA alleviates liver inflammation during non-alcoholic steatohepatitis (NASH) progression by blocking the LPS-TLR4-MyD88 signaling pathway via direct binding to MyD88 and by activation of Nrf2/PPARα signaling [173].

In an α-naphthylisothiocyanate-induced mouse model with cholestatic liver injury, CGA suppresses cell death and neutrophilic and monocytic infiltration and reverses dysregulated hepatocyte transporters and enzymes related to synthesis, uptake, metabolism, and efflux of bile acids [43,174]. In a rat model of hepatic ischemia/reperfusion injury, CGA attenuated liver damage by suppressing HMGB1/TLR-4/NF-κB signaling and mitochondria-mediated apoptosis [175].

### 5.3. Mitigative Effects on Liver Fibrosis and Hepatocellular Carcinoma (HCC) (Figure 1E)

CGA attenuates Schistosoma japonicum cercaria-induced hepatic fibrosis in animals, partially through regulating IL-13/miR-21/Smad7 [176]. CGA suppresses CCl4-induced liver fibrosis by suppressing miR-21/TGF-β1/Smad7 signaling or inhibiting TLR4/NF-κB signaling and stimulating the Nrf2 pathway [22,177,178]. In a methionine and choline deficiency diet (MCDD)-caused nonalcoholic steatohepatitis (NASH) model, CGA increases the biogenetics of mitochondria and suppresses the generation of extracellular matrix triggered by HMGB1 in liver endothelial cells, thus attenuating liver fibrosis [179].

CGA suppresses HepG2 growth and HCC formation via inhibition of ERK1/2, matrix metalloproteinase (MMP)-2/9, and DNA methyltransferase 1 [180,181]. CGA in combination with protocatechuic acid forces HepG2 cells to enter apoptosis [182]. CGA in combination with caffeine and trigonelline can inhibit the tumorigenesis related to diethylnitrosamine (DEN)/CCl4-caused liver fibrosis [183]. CGA can restore the disorganized gut microbiota and aberrant metabolites in DEN/CCl4-caused HCC in animals [184].

### 5.4. Human Subject Studies for Hepatic Protection

In a clinical study with subjects with NDFLD in type 2 DM, neither CGA nor caffeine showed significant effects on improving stiffness of the liver and other hepatic outcomes. The TC was lower in the caffeine group and insulin was higher in the CGA plus caffeine group than in the placebo group, respectively [185]. In a randomized controlled clinical trial with HCC patients (*n* = 291) transcatheter arterial chemoembolization (TACE) therapy was administered, with or without FZJDXJ, a Chinese medicine formulation, for 48 weeks. The active ingredients of FZJDXJ included formononetin, CGA, caffeic acid, luteolin, gallic acid, diosgenin, ergosterol endoperoxide, and lupeol, which might potentially target AKT/CyclinD1/p21/p27 pathways. In addition, molecular docking showed that CGA and gallic acid could effectively interact with the phosphorylation site Thr308 of AKT1. FZJDXJ and TACE treatment significantly prolonged one-year overall survival (OS) and progression-free survival (PFS) of patients compared with TACE treatment alone [186].

## 6. Neuroprotection

CGA has shown diverse neuroprotective effects on various neuropathological conditions which may be exerted through inhibition of neuroinflammation, reduction in ROS production, prevention of oxidation, and suppression of neuronal apoptosis [187,188,189,190].

### 6.1. Protective Effects against Neuronal Injury (Figure 1F)

CGA inhibits H2O2-induced apoptosis by blocking pro-apoptotic factors caspase-3 and pro-poly (ADP-ribose) polymerase (PARP) and upregulating anti-apoptotic factors Bcl-2 and Bcl-X(L) in neuronal cells and PC12 cells [34,191]. CGA can reduce overactive microglia-induced neuroinflammation in the cortex. CGA suppresses TNF-α secretion and NO generation in LPS-stimulated primary microglia, increasing the survival of dopaminergic neurons [192]. CGA counteracts the TNFα-activated NF-kB pathway in an immortalized human oligodendrocyte cell line M03-13 by suppressing intracellular superoxide ions, mitochondrial ROS, and protein levels of NADPH oxidases (NOXs)/dual oxidase 2 (DUOX2) [193]. CGA protects cerebellar granule cells from NO-caused death in vitro [194]. CGA protects rat cortical neurons against glutamate-induced neurotoxicity and oxidation [195] and prevents AMPA-induced neurotoxicity in oligodendrocytes derived from the optic nerve through suppression of PKC and caspase-dependent signaling [196].

CGA and its metabolites are thought to be able to pass the blood–brain barrier (BBB) and execute their impacts on the nervous system [197,198,199,200]. CGA attenuates methotrexate-induced oxidative damage in rat cerebellum [201]. CGA inhibits cadmium-induced rat brain damage via suppressing lipid peroxidation, increasing antioxidant activity, and attenuating mitochondrial dysfunction and DNA breakdown [202]. It has demonstrated its protective effects against scopolamine-induced amnesia in mice [203,204], alcohol-induced neuronal injury in neonates [205], pilocarpine-induced oxidative stress [206], 3-nitropropionic acid-caused neurotoxicity and genotoxicity [207], kainic acid-induced cytotoxicity and learning and memory loss in mice [208], and L-buthionine-(S, R)-sulfoximine-caused oxidation in mouse forebrain [209].

### 6.2. Mitigative Effects on Alzheimer’s Disease (AD) (Figure 1F)

CGA or extracts containing CGA can inhibit Aβ aggregation-caused cellular injury in SH-SY5Y cells, a neuroblastoma cell line, and PC12 cells [210,211,212,213]. It suppresses the Aβ1–42 self-induced aggregation in PC12 cells [213]. In Aβ-treated hippocampal neurons, CGA increases survival and decreases apoptosis via decreasing activities of lactate dehydrogenase (LDH) and the levels of MDA and raising the levels of SOD and GSH-Px [214]. CGA facilitates Aβ clearance and cognitive improvement by enhancing the expression of hippocampal LDL receptor-related protein 1 and restoring perivascular deposition of aquaporin 4 [215].

CGA prevents Aβ deposition and neuronal loss and ameliorates learning and memory deterioration in APP/PS2 mice [216]. CGA restores spatial learning and memory in SAMP8 mice, a mouse model showing plaques with Aβ depositions and age-related cognitive defects [217]. CGA inhibits acetylcholinesterase (AChE) activity in rat brains, suggesting its beneficial effect against cognitive impairment [218,219]. Molecular docking simulations suggest that CGA can bind towards AChE [220]. CGA inhibits AChE, decreases the hippocampal and frontal cortical levels of MDA, and improves the deteriorated short-term or working memory and defective cognition induced by scopolamine, a muscarinic receptor antagonist [203].

### 6.3. Mitigative Effects on Parkinson’s Disease (PD) (Figure 1F)

CGA has demonstrated preventative effects against PD. CGA improves the decrease in α-synuclein-induced cell viability and blocks the interplay between oxidized dopamine and α-synuclein [221]. CGA attenuates the 6-OHDA-caused apoptosis of SH-SY5Y cells [222,223]. CGA combined with caffeic acid prevents rotenone-caused Parkinsonian pathology in nigral dopaminergic and intestinal enteric neurons [224]. CGA enhances the expression of tyrosine hydroxylase and anti-inflammatory cytokine IL-10 and reduces the drug-induced neuroinflammatory factors such as IL-1β, TNF-α, and NF-κB in substantia nigra [192,225]. CGA inhibits the activation of pro-apoptotic proteins including Bax and caspase-3 and elevates the levels of anti-apoptotic factors such as Bcl-2 [226].

### 6.4. Effects on Ischemia-Induced Brain Injury (Figure 1F)

CGA protects against injury caused by cerebral ischemia/reperfusion [227]. It can decrease mortality [228], increase neurological deficit scores [228,229], mitigate sensory–motor functional deficits [198], attenuate infarct volume [198,228,229,230], reduce neuronal loss [231,232,233], suppress brain edema [198,229,230], decrease BBB injury [198,230], and ameliorate ischemia-induced cognitive deficits [229,232,233]. The mechanisms underlying CGA-mediated protection from ischemia-induced brain injury are as follows: (1) It upregulates the activity of SOD2 and GSH and suppresses ROS generation, LDH secretion, and MDA elevation through the Nrf2 pathways [229,232,233]; (2) It decreases the ischemia-induced pro-inflammatory factors such as TNF-α and IL-2 but increases anti-inflammatory cytokines such as IL-4 and IL-13 [230,233]; (3) It inhibits apoptotic markers such as caspase-3 and increases anti-apoptotic factors such as Bcl2 in ischemia [229,230,232]; (4) It facilitates the expression of neurotrophins such as BDNF and NGF for neuronal repair in response to cerebral ischemia/reperfusion [228,229]; (5) It decreases the expression of metalloproteinases such as MMP-2 and MMP-9 for protection of BBB integrity in the cerebral ischemia brain [198]; and (6) It increases endothelial marker CD31 but decreases endothelin-1 to improve from vascular damage [232].

### 6.5. Effects on Cognitive Function (Figure 1F)

CGA protects against anxiolytic and depressive processes in a mouse model of anxiety [234]. CGA improves cognitive impairments in sleep-deprived mice via immunomodulatory effects and gut microbial metabolic modulation. The potential contributive mechanism was Nrf2/PPAR activation [235]. CGA attenuates the polarization of macrophages and alleviates cognitive impairments in an LPS-induced neuroinflammation mouse model by targeting the TNFα signaling pathway [236]. CGA improves memory dysfunction and attenuates frontal cortex inflammation in diabetic rats [237]. Dried loquat fruit extract containing CGA improves corticosterone-induced depression-like behaviors in mice [238].

### 6.6. Modulation of Neuropathic Pain (Figure 1F)

Neuropathic pain is related to immunomodulation and inflammatory response [18]. CGA shows antinociceptive efficacies in pains related to tonic and inflammations and chronic neuropathy [133,239,240,241], which may be a result of CGA’s anti-inflammatory activities on suppression of peripheral release of many pro-inflammatory factors, including TNF-α, NO, and ILs [239,242,243]. Oxidative stress involves all stages of neuropathy and its related pain since free radicals are key mediators causing peripheral nerve injury [244,245]. Data have demonstrated that ROS is a crucial contributor to the development of neuropathic and inflammatory pain [246,247,248,249,250], which can be attenuated by various phenolic antioxidants [251,252,253]. CGA has strong antioxidant activities for scavenging free radicals such as ROS [254]. It is reasonable to posit that CGA can reduce neuropathic pain by scavenging ROS.

CGA-enriched herb extracts execute antinociceptive actions in various animal models [255,256]. Acidosis-induced and trigeminal nociceptive pain can be reduced by CGA [257,258]. CGA can suppress the inflammatory cascade and decrease mechanical and cold hyperalgesia in the rat model of chronic constrictive nerve injury (CCI) [240,241]. The underlying mechanism is probably realized through facilitating the activation of gamma-aminobutyric acid A (GABA_A_) receptors in the spinal cord, a major inhibitory neuronal transmission for pain modulation [259,260] (Figure 2E). However, CGA seems ineffective in mitigating acute pain [133].

CGA may directly act on ion channels related to neuropathic pain for its mitigative effects. For example, voltage-gated potassium channel subfamily A member 4 (Kv1.4), which is specifically expressed in nociceptive sensory neurons in small diameters (A^δ^ and C fibers), is involved in neuropathic pain when its function is suppressed [261,262]. Kv activities are upregulated by CGA in trigeminal ganglions on the basal level and PGE_2_-induced inflammations [263,264], leading to an attenuation of neuronal excitability-related pain induction [264,265,266,267] (Figure 2E). Furthermore, CGA can suppress acid-sensing ion channels in sensory ganglions [257,268], presenting another potential peripheral antinociceptive pathway.

### 6.7. Human Subject Studies for Neuroprotection

Several studies show that regularly prolonged intake of CGA has positive effects on cognitive function in humans [269,270,271]. In a cohort of healthy subjects with self-description of memory decline (*n* = 38, 50–69 years old), individuals were given a CGA-enriched beverage or placebo for 4 months. The data showed that CGA improved some categories of cognitions (such as attention shifting, function of execution, and motor and psychomotor speed) and increased plasma levels of early cognitive impairment biomarkers such as apolipoprotein A1 and transthyretin [270]. In another cohort of the elderly with subjective memory complaints (*n* = 8), subjects were administered CGA (330 mg) for 6 months, and similar improvements were observed including memory for composition and verb use, cognition of flexibility, function of execution and attention, and motor speed. Furthermore, there were reductions in the plasma levels of Aβ42 and Aβ42/Aβ40 and an increase in the plasma level of dehydroepiandrosterone sulfate [271]. In a recent randomized controlled trial on 34 individuals with mild cognitive impairment who were administered two periods of CGA (554 mg of CGA or placebo, twice/day) for 3 months with a monthly interval, data showed improvements in cognitive functions, especially attention and executive function [269]. In a randomized, double-blind, placebo-controlled crossover study, healthy humans with consumption of CGA-enriched coffee berry extracts increased arousal, but limited cognitive effects were observed [272]. Ingestion of CGA (600 mg) over 5 days in healthy subjects (*n* = 9) shortened sleep latency without effects on sleep architecture, enhanced parasympathetic activity, and increased fat oxidation during sleep [273].

## 7. Anticancer Effect

CGA has the role of an anticancer agent in various types of cancer cells by arresting cell proliferation, promoting apoptosis, and facilitating intracellular DNA impairment [13] (Figure 1G).

### 7.1. Breast Cancer

CGA exhibits cytotoxicity in breast cancer cell lines such as MCF-7 with an IC_50_ of 127 µM, resulting in DNA injury, cell cycle stall, and apoptosis [274,275]. A possible mechanism is that CGA can bind to PKC in the cytosol and translocate it to the plasma membrane, thus disturbing the cell cycle, arresting cells at the G1, and reducing cells in the S phase [274]. CGA shows cytotoxicity on breast cancer cell lines such as MDA-MB-231, MDA-MB-453, and 4T1 in dose- and time-dependent manners through downregulation of NF-κB pathway [276]. It also modulates the epithelial–mesenchymal transition (EMT) process of breast cancer cells by downregulation of N-cadherin and upregulation of E-cadherin [276]. In a breast cancer cell-bearing BALB/c mouse model, CGA suppresses tumor growth by increasing the expression of p53, Bax, and the ratio of Bax/Bcl-2 [276,277].

### 7.2. Colorectal Cancer

CGA can stall the cells in the S phase and cause DNA injury in human colon cancer cell lines such as HCT116 and HT29 by increasing ROS production, upregulation of phosphorylated p53, HO-1, and Nrf2 [278]. CGA activates the mitochondrial apoptotic pathway in cancer cells by showing DNA breakdown, cleavage of pro-caspase-9 and PARP-1, and upregulation of Bax and the Bax/Bcl-2 ratio [279]. CGA and its metabolites can increase the levels of pro-caspase-3 and activated caspase-3 in human colon cancer cell lines such as Caco-2 [280]. CGA combined with lactoferrin arrests SW480 cells at the G_0_/G_1_ phase and decreases cell viability [281].

### 7.3. Esophageal Cancer

Evidence reveals that CGA can suppress proliferation and colony formation on many esophageal cancer cell lines such as KYSE30/70/140/150/180/510 [282]. In esophageal cancer cell line-bearing non-obese diabetic (NOD)/severe combined immunodeficiency disease (SCID) mouse models, CGA (50 mg/kg) inhibits the propagation and size of the tumor and reduces esophageal hyperplasia, thus extending mouse lifespan. CGA decreases expressions of survivin and SOX2 in esophageal squamous carcinoma [282].

### 7.4. Leukemia

CGA (10–25 µg/mL) causes the apoptosis of Bcr-Abl^+^ leukemia cell lines by an increase in intracellular H_2_O_2,_ O_2_^−^ and levels of caspases, as well as PARP degradation and suppression of p-STAT-5 and p-CrkL [283]. Similar results have been reported in U937 and HL-60 leukemia cells. CGA (50–200 µM) facilitates cancer cell death through the induction of ROS and activation of caspase-dependent signaling, leading to reduction in membrane potentials of mitochondria, DNA damage, and apoptosis [284,285].

### 7.5. Lung Cancer

CGA (2–50 µM) can suppress the progression of human lung cancer cell line A549 by increasing the levels of annexin-V, Bax, and CASP3, activating p38 and Jun, and decreasing Bcl-2 and tumor stem cell markers including NANOG, POU5F1, and SOX2, indicating multiple kinase pathways and ROS signaling underlying CGA-mediated anti-lung cancer activity [286]. This finding has been echoed by in vivo experiments using an A549-bearing nude mouse (BALB/c) model, in which CGA (120 mg/kg) reduces their tumor mass and size by binding with annexin A2 and inhibiting the expression of NF-κB downstream antiapoptotic genes, thus suppressing cancer cell growth and migration [287].

### 7.6. Melanoma

CGA (1–1.5 mM) reduces the growth of melanoma C32 cells by increasing the expression of antioxidant molecules such as SOD and GSH-Px, thus decreasing oxidation [288]. CGA prevents B16F10 melanoma cell proliferation by facilitating the tumor-associated macrophage (TAM) polarization from M2 to M1. CGA with an anti-PD1 antibody can decrease the CD4^+^ Foxp3^+^ T cell ratio and increase the CD8^+^ T cell ratio, leading to an enhancement of immunotherapeutic activity in vivo [289].

### 7.7. Brain Glioma

CGA (0.5–5 µM) downregulates the macrophagic STAT−1 and STAT-6, leading to the apoptosis and proliferation stall of glioma cells (U87) [290]. In G422 cancer cell-bearing mice, CGA (20 or 40 mg/kg) decreases tumor mass by increasing M1 TAM and suppressing M2 TAM [291]. In glioma C6 cell-bearing Kunming mice, CGA treatment reduces tumor area and prolongs the median survival time of mice [292]. CGA (200 µM) can show neuroprotection of the bortezomib-caused neurite injury and loss of cell volume, which is also confirmed in neuroblastoma SH-SY5Y and rat dPC-12 cells [293].

### 7.8. Osteosarcoma

CGA reduces the proliferation of osteosarcoma cell lines such as U2OS, MG-63, and Saos-2 by increasing the activity of caspase-3, caspase-7, and PARP, and inducing apoptosis through the blockage of the STAT3/Snail pathway [294,295]. CGA in combination with doxorubicin suppresses cellular metabolic activity, colony formation, and cell growth of U2OS and MG-63 cells by upregulating caspase-3 and PARP and suppressing the p44/42 MAPK pathway, thus inducing apoptosis [296].

### 7.9. Pancreatic Cancer

CGA (100–300 µM) can stall cells at the G_2_/M phase and suppress cell proliferation and colony formation of pancreatic carcinoma cells (PANC-1), which can be synergically enhanced in combination with thermal cycling hyperthermia (TC-HT) (10 cycles) with or without a low-intensity pulsed electric field (LIPEF) [297,298]. The underlined mechanism involves CGA-mediated excessive ROS production, causing mitochondrial dysfunction, leading to increases in cleaved levels of caspase-3, caspase-9, PARP, and Bax/Bcl-2 ratio [297,298]. These data are further validated by in vivo experiments showing that CGA can reduce tumor growth and volume in pancreatic cancer cell-bearing nude mice by modifying cancer cell metabolism through decreasing levels of cyclin D1, c-Myc, and cyclin-dependent kinase-2 (CDK-2), interrupting mitochondrial respiration, and suppressing aerobic glycolysis [299].

### 7.10. Prostate Cancer

CGA arrests cells at the phase of G_1_ and inhibits cell viability of prostate cancer cell DU145 by suppressing the levels of HIF-1α and SPHK-1, PCNA, cyclin-D, CDK-4, p-Akt, p-GSK-3β, and VEGF [300].

### 7.11. Renal Cell Carcinoma (RCC)

CGA (IC_50_ 40 µM) selectively suppresses cell proliferation and colony formation of human RCC A498 cells but without effects on human embryonic kidney (HEK293) cells through upregulation of cleaved levels of caspase-3, caspase-9, and PARP and the ratio of Bax/Bcl-2 and inhibition of the PI3K/Akt/mTOR pathway [301].

### 7.12. Human Subject Studies for Cancer Management

In an open-label, dose-escalation phase I trial on patients with recurrent high-grade glioma after standard-of-care treatments (*n* = 26), CGA was intramuscularly injected into patients once daily for 28 days. The median OS after CGA treatment was 11.3 months, which showed a prolonged trend as compared with the median OS (5.7 to 7.5 months) for patients in similar stages under standard-of-care therapeutics [302].

## 8. Skin Protection

CGA has shown diverse dermal protective roles in various skin conditions such as anti-UV-induced photoaging, promoting skin slap survival, improving skin barrier function, mitigating systemic lupus erythematosus (SLE)-like symptoms, and suppressing melanogenesis.

### 8.1. Dermal Protection against Skin Pathologies (Figure 1H)

(1) CGA shows anti-inflammatory and antiaging effects by inhibiting UVA-activated TGF/Smad2/3 signaling, decreasing ROS, pro-inflammatory factors IL-1β and TNF-a, reducing apoptosis and necrosis, attenuating DNA damage, promoting cell repair, and increasing synthesis of collagens in dermal fibroblasts [303,304]; CGA ameliorates deoxynivalenol-induced dermal injury by activating Nrf2 and inhibiting MAPK/NF-kb/NLRP3 pathways [305]; (2) CGA promotes skin flap survival in rats by downregulating MDA and NO, upregulating GSH and SOD, and elevating VEGF expression and capillary density, leading to blood perfusion [306]; (3) CGA restores the epidermal skin barrier by upregulation of filaggrin, involucrin, and envoplakin and induction of diverse responses of cytokines in epidermal keratinocytes [307]; (4) CGA has anti-acne vulgaris effects. CGA rescues *P. acnes*-induced skin lesions in ears including redness, swelling, and erythema, downregulates the levels of pro-inflammatory factors by suppressing NF-κB signaling, and inhibits lipogenesis by attenuating AKT/mTOR/SREBP signaling [308]; and (5) CGA relieves SLE-like skin lesions. CGA down-regulates IL-17 levels, mitigates SLE-caused injuries in the skin and mucous membranes, and improves arthritis-like syndromes in MRL/lpr mice [309]; (6) CGA-containing hydrogel promotes the formation of microvessels from HUVEC cells and proliferation of HaCAT cells. In a skin-wound rat model, CGA hydrogel facilitates the wound-healing process by modulating macrophage polarization, alleviating the production of pro-inflammatory cytokines, enhancing collagen deposition, and increasing the expression of CD31 and VEGF [310].

### 8.2. Anti-Melanogenesis Effects (Figure 1H)

In melanoma B16 cells, CGA likely acts on melanin as a substrate, but its metabolites may inhibit melanogenesis by suppressing tyrosinase activity [311]. CGA and caffeic acid derivatives inhibit melanocyte-stimulating hormone (α-MSH)-induced melanogenesis [312,313,314]. CGA binds to tyrosinase. The molecular docking simulation of CGA on tyrosinase shows the binding energy of −4.59 kcal/mol through interactions with ARG 321 and ARG 374 residues of tyrosinase. Therefore, CGA has the potential as an anti-hyperpigmentation agent through the inhibition of tyrosinase [315].

### 8.3. Human Subject Studies for Skin Protection

In a randomized, double-blind, controlled clinical study, subjects (*n* = 46) were administered jujube syrup containing gallic acid (1140 ± 17.65 μg/mL) and CGA (1520 ± 25.77 μg/mL) or placebo (23 in each group) twice a day for 8 weeks. The number of facial pigment spots and pigmented areas and percentages were significantly lower in the participants taking jujube syrup than in those in the placebo group [316]. In a double-blind, placebo-controlled study, female subjects with mildly xerotic skin (*n* = 49) were given a beverage containing coffee polyphenols (CPPs) (270 mg/100 mL/day) or placebo for 8 weeks. The intake of CPPs improved skin barrier and microcirculatory functions by lowering skin dryness, transepidermal water loss, and skin surface pH, increasing free fatty acids and lactic acid in the stratum corneum, and promoting skin blood flow [317].

## 9. Antiviral and Antimicrobial Effects

CGA exerts diverse functions against pathogen infection and its related inflammation. The viruses in which CGA has shown inhibitory roles include HBV, sowbane mosaic virus, potato virus X, alfalfa mosaic virus, HSV, adenovirus, avian influenza virus, etc. CGA also has shown its anti-bacterial and anti-fungal effects.

### 9.1. Anti-HBV Effects (Figure 1I)

CGA and its metabolites (caffeic acid and quinic acid) exhibit anti-HBV effects. People in northern European countries with greater coffee consumption exhibit a lower rate of HBV infection than the Chinese population, among which the prevalence of HBV infection is approximately 7% [318,319]. Hepatitis B chronic carriers with moderate coffee intake reduce the susceptibility of HCC [320]. The IC50 of CGA on HBV DNA is about 1.2 ± 0.4 μM. The IC50s of caffeic acid on secretion of HBsAg and HBeAg are about 12.7 ± 9.9 μM and 109.3 ± 56 μM, respectively [319,321,322]. CGA mitigates hepatic inflammatory response and fibrotic formation via suppressing TLR-4 signaling [323]. CGA and its hydrolysates can suppress pathogenetic progression toward liver cancer via inhibition of MMP-9, a crucial factor involving the development of human HCC induced by HBV [324,325].

### 9.2. Inhibitory Effects against Other Viruses (Figure 1I)

CGA and its metabolites have anti-viral effects on sowbane mosaic virus, potato virus X, and alfalfa mosaic virus [326]. CGA and caffeic acid show strong inhibitory effects against herpes virus HSV-1 (EC50 = 15.3 μg/mL, SI = 671), HSV-2 (EC50 = 87.3 μg/mL, SI = 118), adenovirus-3 (ADV-3) (EC50 = 14.2 μg/mL, SI = 727), and ADV-11 (EC50 = 13.3 μg/mL, SI = 301) [327]. CGA and its derivatives have effects against avian influenza virus (H5N1) [328]. The cytopathogenic effect (CPE) inhibitory concentration of CGA for HSV-1 in MDBK cells and RNA virus parainfluenza (type-3) (PI-3) in Vero cells is about 3.2 ug/mL [329].

### 9.3. Inhibitory Effects against Bacteria and Fungi (Figure 1I)

The minimal inhibitory concentration (MIC) of CGA ranged from 4 to 16 ug/mL for ATCC stains and from 64 to 128 ug/mL for their corresponding isolated ESµL+ strains (*E. coli*, *P. aeruginosa*, *P. mirabilis*, *K. pneumoniae*, *A. baumannii*, *S. aureus*, *E. faecalis*, and *B. subtilis)* [329]. The MICs of CGA for fungi *C. albicans* and *C. parapsilosis* were 8 and 16 ug/mL, respectively [329].

### 9.4. Anti-Allergic Effect

CGA reduces the allergic response induced by shrimp food in mice, likely by suppressing Acetyl-CoA carboxylase (ACC) and increasing carnitine palmitoyltransferase-1 (CPT-1) and AMPK and ACC phosphorylation [330].

## 10. Extending Lifespan in Worms

CGA reduces the generation of ROS in worms and increases their lifespan through the DAF-16/FOXO and Nrf2/SKN-1 signaling axis under normal conditions or in challenge to oxidation [331]. CGA can prolong about 20.1% of *C. elegans*’ lifespan by attenuating the age-associated decrease in body mobility and enhancing stress challenge via DAF-16-regulated insulin/IGF-1 signaling [332]. CGA prolongs about 24% and 9% of the lifespans of DAF-16a- and DAF-16f-rescued worms, respectively, through the activation of Nrf2/SKN-1 [333] (Figure 1J).

## 11. Other Protective Roles of CGA

### 11.1. Lung Protective Effects

CGA counteracts paraquat-induced oxidative, fibrotic, and inflammatory injuries to the lungs in rats [334]. KAT2A is the crucial regulatory gene for the expression of pro-inflammatory factors. CGA acts as a KAT2A inhibitor, attenuating the acute lung inflammation and improving the impaired respiratory function in a mouse model of LPS-induced acute lung injury [335]. In LPS and polyinosinic:polycytidylic acid (POLY I:C)-induced acute lung injury (ALI)/acute respiratory distress syndrome (ARDS) models, CGA counteracts the inflammatory and oxidative stress in human airway epithelial cells and in BALB/c mice through targeting the TLR4/TLR3/NLRP3 inflammasome axis [336].

### 11.2. Intestinal Protective Effects

CGA alleviates intestinal inflammation and injury in broilers induced by necrotic enteritis challenge through suppressing the mtDNA-cGAS-STING signaling pathway [337]. In a rat model of post-infectious irritable bowel syndrome (PI-IBS), rectal application of CGA ameliorated PI-IBS-related pathologies, probably by increasing glycine levels and modulating gut microbial-released extracellular vesicles [338].

### 11.3. Ovarian Protective Effects

CGA significantly counteracts oxidative stress, pro-inflammatory, and pro-apoptotic markers in cisplatin (CDDP)-induced ovarian damage in rats [339]. CGA mitigates symptoms in patients with polycystic ovarian syndrome (PCOS) and improves follicular development, hormone status, and oxidative stress in PCOS rats, likely through modulating HIF-1alpha signaling [340].

### 11.4. Human Subject Studies for Menopausal Symptom Management

In a randomized, placebo-controlled, double-blind, parallel-group trial with healthy women (*n* = 82), the effects of CGAs on menopausal symptoms were examined. The subjects were administered CGAs (270 mg) or the placebo for 4 weeks. CGAs significantly decreased the modified Kupperman index of menopausal symptoms and reduced the number of hot flushes, the severity of hot flushes during sleep, and the severity of daytime sweats compared to the placebo group. No adverse effects were observed in the CGAs group [341].

## 12. Summary

CGA shows diverse pharmacological effects and acts through multidimensional scientific domains. The mechanistic hubs underlie its integrative functions of anti-inflammation, antioxidation, and modulation of metabolic homeostasis (Figure 1, Table 1). First, CGA can curtail NF-κB, JAK, and MAPK pathways, stalling the production of predominant pro-inflammatory factors including TNF-α, NO, COX-2, PGE2, and ILs. It can contain and thwart inflammatory pathway constituents at multiple levels by counteracting primordial inflammatory factors, attenuating inflammatory propagation, and impeding inflammation-related tissue injury. Second, CGA concurrently elevates multiple pivotal antioxidant factors such as HO-1 and NOQ-1 via Nrf2-dependent or independent pathways, leading to scavenging excessive cellular free radicals. Third, it can regulate and help maintain the metabolic homeostasis of lipids and glucose through the activation of the AMPK pathway, modulating glucose release and absorption and lipid synthesis. Fourth, CGA exhibits neuromodulation by targeting multiple neuroreceptors and channels such as GABA receptors, potassium channels, and acid-sensing ion channels, achieving antinociceptive effects (Figure 2, Table 1).

Though our current understanding of CGA has been substantially expanded in contemporary years, there are many important scientific gaps yet to be addressed in future studies. (1) The specific molecular targets of CGA on NF-κB, MAPK, and Nrf2 pathways remain elusive. Molecular docking modeling can provide an insightful lead to the direct interaction of CGA and targeting molecules, followed by functional characterizations. Furthermore, an in-depth analysis including next-generation sequencing and multiome approaches at tissue and single-cell levels should be applied to reveal a systemic and comprehensive picture of CGA’s biological effects at transcriptional, translational, epigenetic, and intermolecular levels. (2) The pharmacokinetic data of CGA is inadequate due to its limited bioavailability. Upon oral ingestion, a significant portion of CGA remains in the colon and becomes metabolized and absorbed into circulation. Therefore, the observed pharmacological effects are likely to result from CGA and its bioactive metabolites, which further impedes the mechanistic interpretation of the data. Furthermore, efforts to modify the structure of CGA or develop novel and effective drug delivery systems such as liposomes, micelles, and nanoparticles for CGA are ongoing and need further validation for bioavailability, tissue distribution, and efficacy. (3) Clinical studies are required to translate CGA efficacy from bench to bedside for patients. Most current studies are performed on in vitro or in vivo models, which may not truthfully recapitulate the pathological conditions in real patients. Moreover, supraphysiological concentrations of CGA are used in many studies, which may lead to a misinterpretation of the value of CGA effects. (4) There has been a rising popularity of green coffee bean powder recently. The consumption of CGA-enriched natural products such as GCE, fruits, and vegetables at a dose equivalent to daily intake may empower a versatile way to extend its health benefits to the general population without any safety concerns. The development of novel approaches for raw material processing to preserve the CGA and other bioactive substances and improve their release and absorption upon ingestion is a task for the dietary supplementary industry.

In summary, recent advances in our understanding of CGAs have supported its therapeutic potential in many disorders. It is necessary to propel properly designed clinical trials and prospective studies to further elucidate and validate its efficacy in clinics.

## Figures and Tables

**Table 1:** A summary of the potential mechanisms underlying CGA’s pharmacological activities and related experimental models.

Pathological Conditions/Organs	Pharmacological Effects	Experimental Models	Potential Signaling Pathways/Targets	Compound/Natural Sources
Aging	Mitochondria protection and increasing lifespan (Section 10)	*C. elegans* [332,333]	DAF-16-regulated insulin/IGF-1 signaling [332]; activation of Nrf2/SKN-1 [333]	CGA
Cardiovascular system	Hypotensive effect (Section 3.1)	SMCs, SHRs, and cyclosporine-induced hypertensive rats [64,65,66,67]	Elevation of EDRFs, suppression of oxidations and vasoconstrictors, decrease HIF-1α and an enhancement of Shc/Grb2/ERK1/2 signaling [2,64,66,67]	CGA, GCE
		Patients with mild hypertension [7,14,103,104]; patients with borderline or stage 1 hypertension [105]; healthy male adults [106]	Targeting muscarinic acetylcholine receptors [64,65]	CGA; CGA plus HHQ
	Endothelial protection and anti-atherosclerosis (Section 3.2)	EC injuries [75,76,79,80,81]; atherosclerosis and HFD rat [68,69,70]; macrophages and ApoE^−/−^ mice [73,86,87]	Upregulating SIRT1 and AMPK/PGC-1 activity [75] suppressing mtROS/JNK/NF-κB and HIF-1α-VEGF signaling [2,82] regulating A_2A_ receptor/AC/cAMP/PKA pathway [84,85]; activating PPARγ–LXRα–ABCA1 signaling [86,87]	CGA; extract of Crataegus pinnatifida Bge. var. major N.E.Br. fruit
	Cardioprotection (Section 3.3 and Section 3.4)	H9C2 cells; [96] MI animal models induced by ISO, LAD, and CCl_4_, and LAD-induced myocardial I/R SAMP8 mouse [97,98,99,100,101]	Suppressing NF-κB and JNK pathways, regulating PPARα and PI3K/Akt pathways, lower HIF-1α expression, and suppressing cardiac apoptotic signaling [2,90,91,92,96]	CGA; CGAs-enriched chrysanthemum extracts; CGA-enriched extracts from Lindl. Benth.
		healthy adults [108,109,110,111,112,113,114]; subjects with metabolic syndrome [115,116]	n.a.	CGA-enriched coffee beverages; nutraceutical containing CGA and luteolin extracts; GCE
Inflammation and oxidation	Anti-inflammatory and oxidative effects (Section 2.1 and Section 2.3)	Macrophage [32]; 3T3-L1 cells [27]; carbon tetrachloride or acetaminophen-induced liver injury in mice [20,23,24] Weaned Pigs, LPS-induced mice, I/R rat liver injury, endotoxic shock-induced acute liver injury [28,30,31,33]	Suppressing TLR4, TNF-α, NF-κB, and MAPK pathways [28,29,30,31]; activation of CD36/AMPK/PGC-1α [32] and Nrf2 signaling [20,34,35,36,37]	CGA; Taraxacum officinale root
		Healthy postmenopausal women [55]; healthy subjects [56]	n.a.	Bioactive yogurt containing curcumin and CGA; polyphenol-rich beverage
Liver	Protection of the liver from injuries (5.1)	Metals, chemicals, and toxins: sodium arsenite [137], Pb [138], Cd [139], aluminum chloride [140], polychlorinated biphenyls [141], TAA [142], CCl4 [143], D-gal [144], L-carnitine [145], LPS [146,147], palmitic acid [148], and aflatoxin B1 [149]	Activating the Nrf2 pathway, promoting mitophagy, and suppressing the TLR4/NF-κB pathway [20,24,150,151,152,153,154,155,156,157,158,159]	CGA
	Decreasing NAFLD injury (Section 5.2 and Section 5.4)	Hepatic cell line HepG2 [162,163]; HFD mice [123,164,165]; α-naphthylisothiocyanate-induced mouse model with cholestatic liver injury [43,174]; rat model of hepatic ischemia/reperfusion injury [175]	Inhibiting JNK signaling [123,164,165]; blocking the LPS-TLR4-MyD88 signaling pathway and Nrf2/PPARα signaling [173]; suppressing sphingosine kinase 1/sphingosine-1-phosphate/TLR4 pathways [171]; suppressing HMGB1/TLR-4/NF-κB and mitochondria-mediated apoptosis [175]	CGA, CGA in combination with metformin or geniposide or telmisartan
		Human subjects with NDFLD in type 2 DM [185]	n.a.	CGA, CGA plus coffeine
	Mitigation of liver fibrosis and HCC (Section 5.3 and Section 5.4)	Schistosoma japonicum cercaria-induced hepatic fibrosis [176]; CCl4-induced liver fibrosis [22,177,178]; DEN/CCl4-caused HCC [184]; diethylnitrosamine (DEN)/CCl4-caused liver fibrosis [183]	Regulating IL-13/miR-21/Smad7 [176]; suppressing miR-21/TGF-β1/Smad7 signaling or inhibiting the TLR4/NF-κB signaling and stimulating the Nrf2 pathway [22,177,178]	CGA; CGA with protocatechuic acid; CGA plus caffeine and trigonelline
		HCC patients [186]	Targeting AKT/CyclinD1/p21/p27 pathways [186]	FZJDXJ (formononetin, CGA, caffeic acid, luteolin, gallic acid, diosgenin, ergosterol endoperoxide, and lupeol)
Glucose and lipid metabolism	Metabolic homeostasis modulation (Section 2.2 and Section 2.3)	HFD mice [52]; HFD golden hamsters or rats [50,51]	Activation of AMPK pathways [4]	CGA; GCE
		Patients with IGT [57]; healthy subjects [58,61]; overweight dyslipidemic subjects [59]; hypercholesterolemic subjects [60]; overweight subjects [62]; patients with metabolic syndrome [8]	n.a.	CGA; cooked ham (22.5 mg CGA/100 g cooked ham); nutraceutical (containing bergamot, phytosterols, vitamin C, and CGA); green/roasted (35:65) coffee; coffee; GCE
Nervous system	Protection of neuronal injury (Section 6.1)	Neuronal cells and PC12 cells [34,191]; oligodendrocyte [196] and OL cell line M03-13 [193]. granule cells [194]; rat cortical neurons [195]	Suppression of TNFα/NF-kB [193] and PKC and caspase-dependent signaling [196]	CGA
	Neuronal protection in AD and PD (Section 6.2 and Section 6.3)	SH-SY5Y cells and PC12 cells [210,211,212,213]; APP/PS2 mice [216]; SAMP8 mice [217]; molecular docking [220]	Inhibition and binding to AChE [218,219,220]; blockage of the interplay between oxidized dopamine and α-synuclein [221]; anti-apoptotic pathways [226]	CGA; CGA combined with caffeic acid
	Decreasing ischemia-induced brain injury (Section 6.4)	Cerebral I/R rat models [229,232,233]	Activation of Nrf2 pathways [229,232,233]; Inhibition of the TNF-α pathway [230,233] and the apoptotic pathway [229,230,232]; decrease in the expression of metalloproteinases such as MMP-2 and MMP-9 [198]	CGA
	Cognitive improvement (Section 6.5 and Section 6.7)	Mouse model of anxiety [234]; sleep-deprived mice activation [235]; LPS-induced neuroinflammation mouse model [236]; diabetic rats [237]; corticosterone-induced depression-like mice [238]	Activation of Nrf2/PPAR [235]; inhibition of the TNFα signaling pathway [236]	CGA
		Healthy subjects with mild cognitive impairment [269,270,271]; healthy subjects observed [272,273]	n.a.	CGA; CGA-enriched coffee berry extracts
	Neuropathic pain (Section 6.6)	A chronic inflammatory pain model of mice and carrageenan-induced rat hind paw edema [255,256]; rat model of CCI [240,241]; trigeminal ganglion inflammations [263,264] sensory ganglions [257,268]	Suppression of peripheral release of pro-inflammatory factors, including TNF-α, NO, and ILs [239,242,243]; activation of GABA_A_ receptors [259,260]; suppression of Kv channels [263,264] and acid-sensing channels [257,268]	Mansoa alliacea extracts; Cheilanthes farinose extracts; CGA
Pancreas and DM	Protecting β-cells and improving β-cell function (Section 4.1)	β cells and Langerhans from rat islets [119,120]; mice fed on HFD or high-fat milk, spontaneously obese mice, or rats fed on HFD [121,122,123]; STZ-induced DM rats [126]	Anti-oxidative stress [125] and anti-inflammatory response [126]	CGA; CGA with tetrahydrocurcumin
	Mitigation of DM complications (Section 4.2)	A diabetic nephropathy rat model [127,128]; DM mice [129,130]; diabetic DPN DM mice [132]	Anti-oxidative and anti-inflammatory response [127,128,129,130,132]	CGA; CGA-enriched extracts
	Glycemic control in human subjects (Section 4.3)	Healthy human subjects [134]; subjects with IFG [136]; patients with metabolic syndrome [8]	n.a.	GTC together with coffee CGA; CGA-rich Cs extracts; GCE
Pathogen infections	Anti-viral effects (9.1)	Sowbane mosaic virus, potato virus X, and alfalfa mosaic virus [326]; RSV, HSV-2, ADV-3, ADV-11 [327]; H5N1 [328]; HSV-1 in MDBK cells and in Vero cells [329]	n.a.	CGA
	Anti-bacterial effects (Section 9.2)	*A. baumannii*, *B. subtilis*, *E. coli*, *E. faecalis*, *K. pneumoniae*, *P. mirabilis*, *P. aeruginosa*, and *S. aureus* [329]	n.a.	CGA
	Anti-Fungal effects (Section 9.3)	*C. albicans* and *C. parapsilosis* [329]	n.a.	CGA
	Anti-allergic effects (Section 9.4)	Shrimp food-fed mice [330]	Increase in CPT-1 and AMPK and ACC phosphorylation [330]	CGA
Skin	Dermal protection (Section 8.1)	Dermal fibroblasts [303,304]; skin flap survival in rats [306]; epidermal keratinocytes [307]; MRL/lpr mice [309]	Inhibition of MAPK/NF-kb/NLRP3 pathways [305] and KT/mTOR/SREBP signaling [308]	CGA
	Anti-melanogenic effects (Section 8.2)	Molecular docking simulation and in vitro kinetic assay [312,313,314,315]	Inhibition of α-MSH [312,313,314]; inhibition of tyrosinase [315]	CGA
	Protection of skin barrier and improvement in microcirculation (Section 8.3)	Human female subjects with mildly xerotic skin [317];	n.a.	Beverage containing coffee polyphenols
Tumor	Inhibiting tumor cell proliferation/increasing chemo-sensitivity (Section 7.1, Section 7.2, Section 7.3, Section 7.4, Section 7.5, Section 7.6, Section 7.7, Section 7.8, Section 7.9, Section 7.10 and Section 7.11)	Breast cancer cell line MCF-7 [274,275]; colon cancer cell lines HCT116, HT29 [278], Caco-2 [280]; esophageal cancer cell lines KYSE30/70/140/150/180/510 [282]; leukemia cell lines U937, and HL60 [284,285]; lung cancer cell line A549 [287]; melanoma C32 and B16F10 [288,289]; glioma cells U87 [290]; osteosarcoma cell lines U2OS, MG-63, and Saos-2 [294,295]; pancreatic carcinoma PANC-1 [297,298]; prostate cancer cell DU145 [300]; and RCC A498 cells [301]; tumor-bearing SCID mouse models [282,287]	Increase in p53, Bax, and the ratio of Bax/Bcl-2 [276,277]; blockages of (1) p-STAT-5 and p-CrkL [283,287], (2) the NF-kb pathway [276], (3) the STAT3/Snail pathway [294,295], (4) the PI3K/Akt/mTOR pathway [301], and EMT [276]	CGA
	Cancer management in patients (Section 7.12)	Patients with recurrent high-grade glioma [302]	n.a.	CGA
Others	Lung injury protection (Section 11.1)	LPS-induced acute lung injury mouse model [335]; LPS/POLY I:C-induced ALI/ARDS in human epithelial cells [336]	KAT2A inhibitor [335]; targeting of the TLR4/TLR3/NLRP3 inflammasome axis [336]	CGA
	Intestinal protection (Section 11.2)	Broilers induced by necrotic enteritis challenge [337]; a rat model of PI-IBS [338]	Suppression of the mtDNA-cGAS-STING signaling pathway [337]; modulation of gut microbial-released extracellular vesicles [338]	CGA
	Ovarian protection (Section 11.3)	CDDP-induced ovarian damage in rats [339]; PCOS rats [340]	HIF-1alpha signaling [340]	CGA
	Menopausal symptom management (Section 11.4)	Human, healthy women [341]	n.a.	CGA

n.a., no data available.
